# Generalized Dystonia With Cervical Predominance in a Patient With a *De Novo TCF20* Variant

**DOI:** 10.1002/acn3.70534

**Published:** 2026-10-09

**Authors:** Melisa Kilic, Petr Dusek, Ibrahim Kamaci, Bahar Gulmez, Gul Yalcin Cakmakli, Ersin Tan, Sibel Ugur Iseri, Zuhal Yapici

**Affiliations:** ^1^ Department of Genetics Aziz Sancar Institute of Experimental Medicine, Istanbul University Istanbul Türkiye; ^2^ Institute of Graduate Studies in Health Sciences Istanbul University Istanbul Türkiye; ^3^ Department of Neurology Charles University 1st Faculty of Medicine Prague Czech Republic; ^4^ Department of Neurology Istanbul University Faculty of Medicine Istanbul Türkiye; ^5^ Department of Neurology Hacettepe University Faculty of Medicine Ankara Türkiye

**Keywords:** dystonia, frameshift variant, hyperkinetic movement disorder, *TCF20*

## Abstract

*TCF20* has been implicated in developmental delay and intellectual disability, with dystonia reported less frequently. We describe a woman in her early twenties with childhood‐onset learning difficulties, progressive dysarthria, tremor, and generalized dystonia with marked cervical predominance, associated with a *de novo* *TCF20* frameshift variant identified by whole genome sequencing. Quantitative brain MRI demonstrated numerically increased globus pallidus susceptibility and reduced volume compared with controls, without clear evidence of iron accumulation. This case further supports generalized dystonia as a recognizable manifestation of *TCF20*‐related disorders and suggests a possible link between *TCF20* dysfunction and globus pallidus involvement in dystonia pathophysiology.

## 1 Introduction

*TCF20* encodes a transcriptional co‐regulator involved in neurogenesis, brain development, and neuronal function through regulation of genes associated with dendritic and synaptic architecture [1, 2]. Heterozygous *TCF20* variants are associated with *TCF20*‐associated neurodevelopmental disorder (TAND), typically characterized by developmental delay (DD), intellectual disability (ID), and behavioral abnormalities [3]. Autism spectrum traits, attention‐related disorders, hypotonia, structural brain abnormalities, and facial dysmorphism have also been reported [3].

Although TAND is primarily recognized as a neurodevelopmental condition, some patients exhibit motor manifestations, including unsteady gait, tremor, or coordination difficulties [3]. Dystonia has only recently emerged as a potential phenotypic feature and remains rarely reported [4, 5].

Here, we present a patient with a *de novo* *TCF20* variant whose phenotype was dominated by dystonia, further expanding the clinical spectrum of TAND.

## 2 Case

A 23‐year‐old woman presented with generalized dystonia with marked cervical predominance. Her parents were non‐consanguineous, and the family history was unremarkable. Since early childhood, she had exhibited unclear speech, which became more apparent around preschool age. At age eight, she began to develop right upper limb posturing with semiflexion of the wrist and elbow and occasionally dragged her right foot while walking. Neurocognitive evaluation performed because of learning difficulties confirmed mild intellectual disability. At age nine, similar symptoms emerged on the left side and gradually progressed. From age 15, she required walking support and developed cervical dystonia. At 17, jaw contractions impaired chewing of solid foods. From age 19 onward, her speech progressively deteriorated and eventually became incomprehensible.

Neurological examination revealed marked dysarthria, hypophonia, diminished palatal reflexes, and restricted tongue movement. Ocular pursuit and saccades were normal. Bradykinesia and reduced facial expression were noted. Muscle tone examination showed spasticity in the lower limbs and cogwheel rigidity in the upper limbs, particularly at the right wrist. Dystonia predominated in the cervical region, with anterocollis and right‐sided laterocollis, along with truncal dystonia with rightward lateroflexion (Segment 1 in Video S1). Jaw‐closing dystonia was evident. Milder dystonic contractions, mainly of the flexors, were noted in the extremities. Bilateral, left‐predominant rest and postural tremor was accompanied by titubation. Deep tendon reflexes were brisk bilaterally, including elicitable pectoral reflexes, and bilateral Achilles clonus was noted. Spontaneous bilateral dorsiflexion of the great toes was observed. The Myerson sign was positive, while Hoffmann and palmomental reflexes were negative. Mild dysmetria was present, and she could not tandem walk, likely due to lower limb dystonia.

Romberg's test was negative, although postural instability was present. She could still take a few unsupported steps, but her dystonic gait showed visible contractions in both feet (more prominent on the right), left shoulder elevation, absent arm swing, semiflexed upper limb posturing, and rightward truncal lateroflexion (Segment 2 in Video S1). Cognitive evaluation demonstrated preserved basic attention but marked deficits in sustained and executive attention and working memory, suggestive of frontal dysfunction. Mild‐to‐moderate memory impairment appeared secondary to attentional deficits, with preserved encoding. Visual perception of static objects (ventral stream, right occipitotemporal predominance) was severely impaired. Confrontation naming and spatial–temporal orientation were preserved. The Mini‐Mental State Examination score was 23/30.

Routine laboratory tests, cerebrospinal fluid analysis, electroencephalography, and electromyography were unremarkable. Fundoscopy and optical coherence tomography were normal. On cranial 3‐Tesla MRI, the globus pallidus appeared relatively hypointense bilaterally on T2‐weighted and susceptibility‐weighted imaging (SWI). Cranial computed tomography was unremarkable. To further evaluate possible basal ganglia iron accumulation, quantitative susceptibility mapping (QSM) and volumetry of deep gray matter nuclei were performed and compared with data from five age‐ and sex‐matched controls. QSM and T1‐weighted images were processed using a cloud‐based multi‐atlas automated gray matter nuclei segmentation platform [6]. In the globus pallidus, magnetic susceptibility values were bilaterally numerically higher (particularly in the external segment) and volumes lower compared with controls; however, given the single‐patient design and variability among controls, no formal statistical inference can be drawn from these exploratory findings (Figure 1).

**FIGURE 1:**
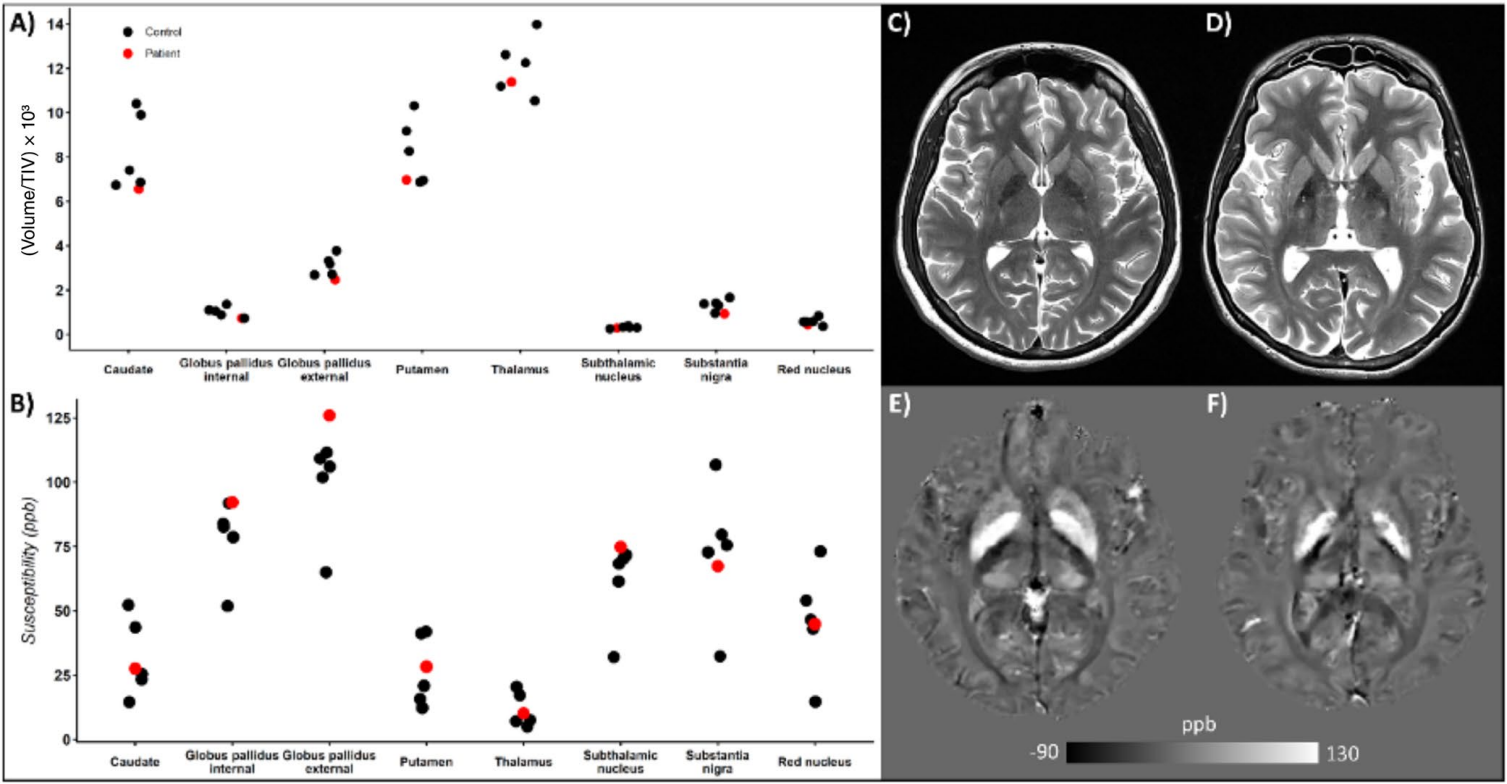
Brain MRI analysis. Dot plots showing (A) regional volumes and (B) mean bulk regional magnetic susceptibility of deep gray matter nuclei in the patient (red circles) and five healthy controls (black circles). Regional volumes are normalized to total intracranial volume (TIV); raw magnetic susceptibility values are reported in parts per billion (ppb). Individual data points are shown to emphasize the exploratory nature of this single‐patient comparison; no statistical inference is intended. T2‐weighted images of (C) a healthy control (age 25 years) and (D) the patient, both acquired on the same 3T scanner using identical turbo spin‐echo sequence parameters (echo time = 104 ms, repetition time = 5250 ms) and displayed with identical windowing. Quantitative susceptibility maps of (E) the healthy control and (F) the patient.

The patient underwent a 3‐month levodopa trial with gradual dose escalation from 62.5  mg/day to a maximum dose of 250 mg/day, without clear benefit. Trihexyphenidyl was titrated to 18 mg/day without satisfactory response. She is currently managed with periodic botulinum toxin injections, providing partial relief of cervical and jaw‐closing dystonia.

## 3 Genetic Analyses

Written informed consent was obtained from the proband and her parents in accordance with the study protocol approved by the Clinical Ethics Committee of Istanbul University, Istanbul Faculty of Medicine. Research‐based whole genome sequencing (WGS) was performed for the proband on the Illumina NovaSeq platform. Variants were analyzed using the GenomizeSEQ pipeline (hg38). Study‐specific filtering prioritized high‐ and moderate‐impact protein‐altering variants according to Ensembl consequence categories. Homozygous and compound heterozygous variants with a minor allele frequency (MAF) < 0.5% and heterozygous variants with MAF < 0.1% were evaluated. Exonic, splicing, and untranslated region variants were analyzed with a focus on genes annotated to the HPO term Dystonia (HP:0001332), and the same gene set was screened for coding‐region structural variants using the GenomizeSEQ SV analysis interface.

Our analysis identified a heterozygous *TCF20* variant, ENST00000677622.1 (NM_001378418.1):c.932_933del; p.Gln311ArgfsTer22, which was not detected in either parent by Sanger sequencing and was therefore consistent with a *de novo* event (Figure 2); parentage was not formally confirmed. The variant was absent from population databases, including gnomAD (v4.1.0) and the RGC Million Exome Variant Browser, but has been reported in ClinVar (Accession: VCV000599637.4). It introduces a premature stop codon in exon 2 of the *TCF20* gene, which is the first coding exon and unusually large, spanning 5691 base pairs. The variant is predicted to result in loss of function, a known pathogenic mechanism in TAND. According to ACMG criteria, it is classified as pathogenic (PVS1, PM2, PS4_Moderate, and PM6).

**FIGURE 2:**
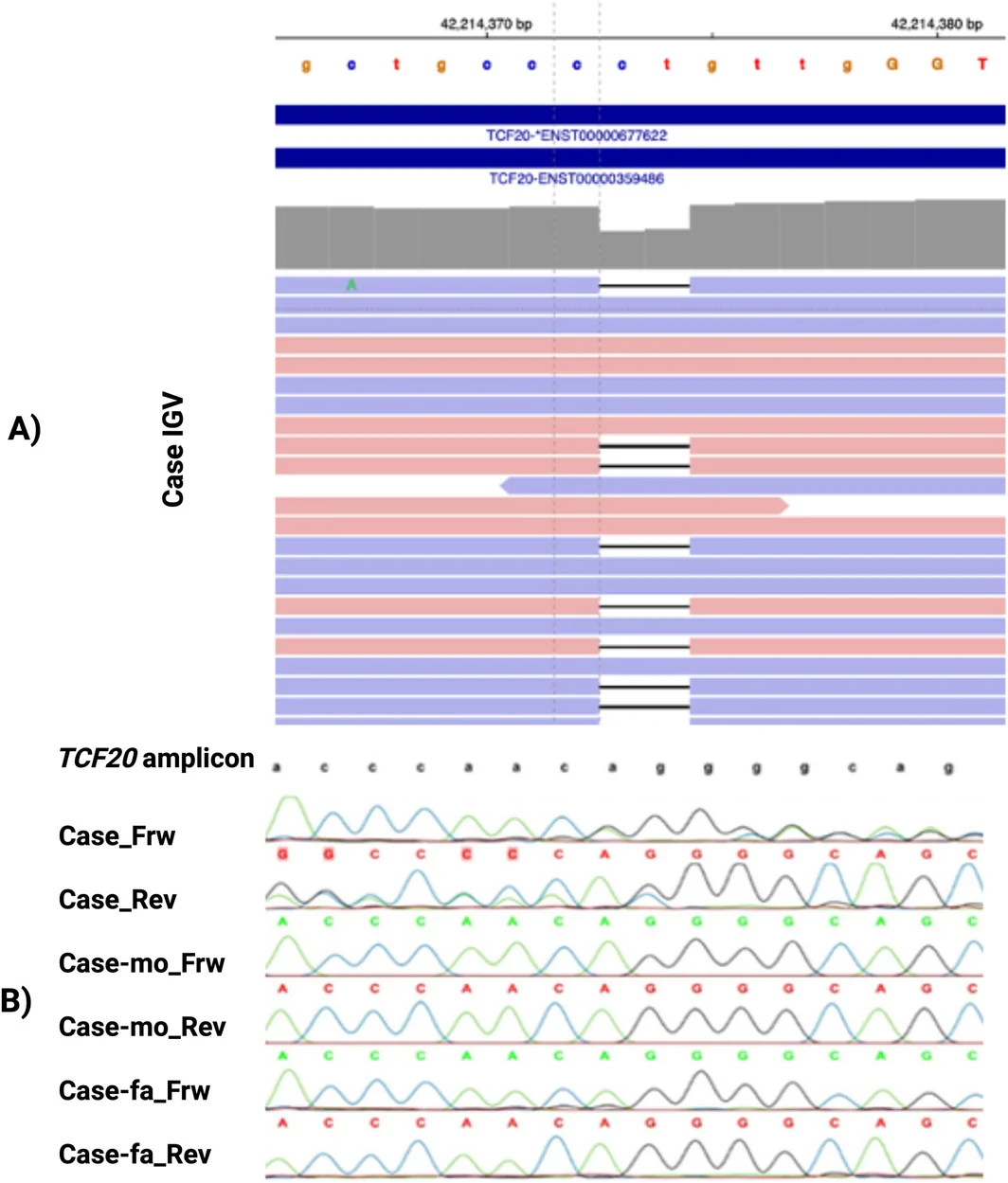
Genetic findings in the case with a *de novo* *TCF2* *0* variant. (A) Integrative Genomics Viewer (IGV) visualization of the *TCF20* variant ENST00000677622.1:c.932_933del (p.Gln311ArgfsTer22) based on the hg38 reference genome. (B) Sanger sequencing segregation analysis demonstrated that the variant was absent in both parents, consistent with a *de novo* occurrence. fa, father; Frw, forward sequence; mo, mother; Rev, reverse sequence.

## 4 Discussion

Advances in understanding the molecular partners of TCF20 have expanded its phenotypic spectrum. A recent study demonstrated a direct interaction between TCF20 and MeCP2, a protein implicated in Rett syndrome [7]. Both proteins are highly co‐expressed in neurons and regulate neuronal gene expression. This molecular link may explain overlapping neurodevelopmental features in *TCF20*‐ and *MECP2*‐related disorders, including the frequent association with autism spectrum disorder. Motor disturbances reported in Rett syndrome, such as tremor and ataxia, have also been described in TAND [3, 8], although movement disorder subtypes were not further characterized. Hyperkinetic movement disorders, particularly dystonia, are increasingly recognized in patients with *TCF20* loss‐of‐function (LOF) variants [4, 5]. Svorenova et al. [4] reported two patients with previously unrecognized dystonia: one with a progressive spastic‐ataxic‐dystonic syndrome with levodopa‐unresponsive generalized dystonia and myoclonus, and another with childhood‐onset toe‐walking followed by generalized dystonia. These cases suggest that hyperkinetic movement disorders, particularly dystonia, are an underrecognized component of the TAND phenotype. Similarly, Prasun et al. [5] described an adolescent initially diagnosed with dystonic cerebral palsy who was later found to carry a *de novo* *TCF20* LOF variant after developing fluctuating spastic‐dystonic lower limb features. More recently, Magistrelli et al. [9] reported a 70‐year‐old man with a *TCF20* variant who developed craniocervical dystonia and upper limb myoclonus in midlife. Collectively, these observations suggest that dystonia, whether progressive, fluctuating, or late‐onset, represents a diagnostically important yet previously underestimated manifestation of TAND.

Previous studies have reported predominantly normal or inconspicuous brain MRI findings in TAND. When present, abnormalities were nonspecific and included atypical corpus callosum morphology, mild white matter gliosis, Chiari malformation, or cerebellar hypoplasia, without a consistent or disease‐specific imaging pattern [3]. QSM has increasingly been used to investigate susceptibility changes and pathogenic mechanisms in neurological disorders, including movement disorders, particularly neurodegeneration with brain iron accumulation (NBIA), as well as neurodevelopmental conditions [10, 11]. In the present case, QSM was performed to further characterize the neuroimaging phenotype in early‐onset progressive dystonia and equivocal SWI signal changes. Although bilateral globus pallidus susceptibility values were numerically higher (particularly in the external segment) and volumes lower than those of controls, variability among controls and the single‐patient design preclude formal statistical inference. Hence, these findings cannot confirm or exclude an NBIA‐related mechanism and should be considered exploratory only.

The patient's generalized dystonia with cervical predominance supports the contribution of dystonia to the TAND spectrum. This case underscores the importance of detailed clinical phenotyping in guiding genetic diagnosis and supports inclusion of *TCF20* in relevant gene panels. The same *TCF20* variant identified in our patient has previously been reported in two unrelated female patients [3, 12]. In the first case, phenotypic information was collected through clinician questionnaires, which may have led to underrecognition of hyperkinetic movement disorders; notably, dystonia was not described. The second was identified through systematic reanalysis within Solve‐RD, a large collaborative European rare disease reanalysis initiative. In that case, generalized dystonia was noted without detailed motor characterization. Shared features across reports include dysarthria, tremor, ataxia, and intellectual disability (Table S1).

This study is limited by its single‐case design, the small control sample, and the lack of functional validation. Although QSM was used for phenotypic characterization, the findings remain exploratory and were not intended for definitive patient–control comparisons. Nevertheless, the overall phenotype raises the possibility of a link between *TCF20* dysfunction, globus pallidus alterations, and dystonia pathophysiology, warranting further investigation in larger cohorts.

## Author Contributions

Z.Y. and S.U.I. conceived and designed the study. Z.Y. and S.U.I. also provided critical revisions of the manuscript. S.U.I. and M.K. conducted the bioinformatics analyses, variant annotation, and data interpretation. M.K. performed the laboratory experiments, including DNA extraction and validation assays, and also prepared the draft manuscript together with I.K. The brain MRI analyses were conducted by P.D. and I.K., who were responsible for the interpretation of the QSM data. B.G., G.Y.C., E.T., I.K., P.D., and Z.Y. were responsible for patient recruitment, clinical evaluation, and collection of clinical data. All authors contributed to the drafting and editing of the manuscript, and all authors read and approved the final version.

## Funding

This study was supported by the TÜBİTAK 2210‐A program, Scientific Research Projects Coordination Unit of Istanbul University (Project No: TYL‐2024‐40633) and the ISTisNA Project (ISTKA No: TR10/22/THN).

## Conflicts of Interest

The authors declare no conflicts of interest.

## Supporting information


**Table S1:** Clinical characteristics of the current patient compared to previously described cases with the same *TCF20* variant.

**Video S1:** Phenomenological features of dystonia in the case.

## Data Availability

The data supporting the findings of this study are available from the corresponding author upon reasonable request. Due to patient privacy and ethics restrictions, raw sequencing data are not publicly available.
